# Methyl (*E*)-3-(2-bromo-4,5-dimeth­oxy­benzyl­idene)dithio­carbazate

**DOI:** 10.1107/S1600536811039304

**Published:** 2011-10-12

**Authors:** Zheng Fan, Yan-Lan Huang, Fang Tong, Yi-Wei Huang, Shang Shan

**Affiliations:** a College of Biological and Environmental Engineering, Zhejiang University of Technology, People’s Republic of China; b College of Chemical Engineering and Materials Science, Zhejiang University of Technology, People’s Republic of China

## Abstract

The title compound, C_11_H_13_BrN_2_O_2_S_2_, was obtained from the condensation reaction of methyl dithio­carbazate and 2-bromo-4,5-dimeth­oxy­benzaldehyde. In the mol­ecule, the benzene ring and dithio­carbazate fragment are located on opposite sides of the C=N bond, showing an *E* conformation. The dithio­carbazate fragment is approximately planar (r.m.s deviation = 0.0281 Å) and the mean plane is oriented at a dihedral angle of 11.38 (15)° with respect to the benzene ring. In the crystal, pairs of N—H⋯S hydrogen bonds link the mol­ecules into centrosymmetric dimers.

## Related literature

For applications of hydrazone and its derivatives in the biological field, see: Okabe *et al.* (1993 ▶); Hu *et al.* (2001 ▶). For related structures, see: Shan *et al.* (2008*a* ▶,*b* ▶,*c* ▶). For the synthesis, see: Hu *et al.* (2001 ▶).
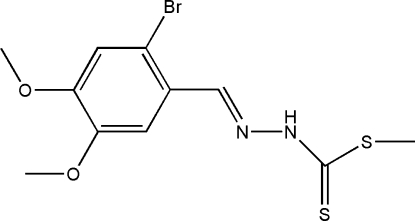


## Experimental

### 

#### Crystal data

C_11_H_13_BrN_2_O_2_S_2_
*M* *_r_* = 349.26
Triclinic, 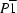

*a* = 5.2460 (12) Å
*b* = 11.781 (5) Å
*c* = 12.400 (5) Å
α = 102.347 (3)°
β = 100.930 (4)°
γ = 101.874 (4)°
*V* = 710.4 (4) Å^3^
*Z* = 2
Mo *K*α radiation
μ = 3.18 mm^−1^
*T* = 293 K
0.42 × 0.28 × 0.25 mm

#### Data collection

Rigaku R-AXIS RAPID IP diffractometer
Absorption correction: multi-scan (*ABSCOR*; Higashi, 1995 ▶) *T*_min_ = 0.73, *T*_max_ = 0.82
5185 measured reflections
2553 independent reflections
2051 reflections with *I* > 2σ(*I*)
*R*_int_ = 0.026

#### Refinement

*R*[*F* ^2^ > 2σ(*F* ^2^)] = 0.035
*wR*(*F* ^2^) = 0.079
*S* = 1.02
2553 reflections
166 parameters
H-atom parameters constrained
Δρ_max_ = 0.32 e Å^−3^
Δρ_min_ = −0.35 e Å^−3^

### 

Data collection: *PROCESS-AUTO* (Rigaku, 1998 ▶); cell refinement: *PROCESS-AUTO*; data reduction: *CrystalStructure* (Rigaku/MSC, 2002 ▶); program(s) used to solve structure: *SIR92* (Altomare *et al.*, 1993 ▶); program(s) used to refine structure: *SHELXL97* (Sheldrick, 2008 ▶); molecular graphics: *ORTEP-3 for Windows* (Farrugia, 1997 ▶); software used to prepare material for publication: *WinGX* (Farrugia, 1999 ▶).

## Supplementary Material

Crystal structure: contains datablock(s) I, global. DOI: 10.1107/S1600536811039304/xu5332sup1.cif

Structure factors: contains datablock(s) I. DOI: 10.1107/S1600536811039304/xu5332Isup2.hkl

Supplementary material file. DOI: 10.1107/S1600536811039304/xu5332Isup3.cml

Additional supplementary materials:  crystallographic information; 3D view; checkCIF report

## Figures and Tables

**Table 1:** Hydrogen-bond geometry (Å, °)

*D*—H⋯*A*	*D*—H	H⋯*A*	*D*⋯*A*	*D*—H⋯*A*
N2—H2*N*⋯S1^i^	0.86	2.62	3.456 (4)	166

Symmetry code: (i) 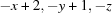.

**Fig. 1.**
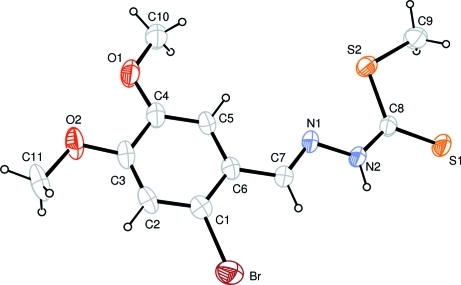
The molecular structure of the title compound with 40% probability displacement (arbitrary spheres for H atoms).

## supplementary crystallographic information

### Comment

Hydrazone and its derivatives have shown the potential application in the biological field (Okabe *et al.*, 1993; Hu *et al.*, 2001). As part of the ongoing investigation on anti-cancer compounds, the title compound has recently been prepared in our laboratory and its crystal structure is presented here.

In the molecules, the benzene ring and dithiocarbazate fragment are located on the opposite sides of the C═N bond, showing the E-configuration. This agrees with those found in the structures reported previously (Shan *et al.*, 2008*a*,*b*). The dithiocarbazate fragment is approximately planar, the r.m.s deviation being 0.0281 Å; its mean plane is oriented with respect to the benzene ring at 11.38 (15)°, similar to that found in a related structure (Shan *et al.* 2008*c*). In the crystal structure, intermolecular N—H···S hydrogen bonding links molecules to form the centro-symmetric dimers (Table 1).

### Experimental

Methyl dithiocarbazate was synthesized as described previously by Hu *et al.* (2001). Methyl dithiocarbazate (0.24 g, 2 mmol) and 2-bromo-4,5-dimethoxybenzaldehyde (0.49 g, 2 mmol) were dissolved in ethanol (20 ml), then acetic acid (0.2 ml) was added to the ethanol solution with stirring. The mixture solution was refluxed for 6 h. After cooling to room temperature, microcrystals appeared. The microcrystals were separated from the solution and washed with cold water three times. Recrystallization was performed twice with absolute methanol to obtain colourless single crystals of the title compound.

### Refinement

H atoms were placed in calculated positions with C—H = 0.93–0.96 Å and N—H = 0.86 Å, and refined in riding mode with *U*_iso_(H) = 1.5*U*_eq_(C) for methyl H atoms and 1.2*U*_eq_(C,N) for the others.

### Crystal data

**Table:**

C_11_H_13_BrN_2_O_2_S_2_	*Z* = 2
*M**_r_* = 349.26	*F*(000) = 352
Triclinic, *P*1	*D*_x_ = 1.633 Mg m^−^^3^
Hall symbol: -P 1	Mo *K*α radiation, λ = 0.71073 Å
*a* = 5.2460 (12) Å	Cell parameters from 2553 reflections
*b* = 11.781 (5) Å	θ = 2.8–25.2°
*c* = 12.400 (5) Å	µ = 3.18 mm^−^^1^
α = 102.347 (3)°	*T* = 293 K
β = 100.930 (4)°	Prism, colorless
γ = 101.874 (4)°	0.42 × 0.28 × 0.25 mm
*V* = 710.4 (4) Å^3^	

### Data collection

**Table:**

Rigaku R-AXIS RAPID IP diffractometer	2553 independent reflections
Radiation source: fine-focus sealed tube	2051 reflections with *I* > 2σ(*I*)
graphite	*R*_int_ = 0.026
Detector resolution: 10.0 pixels mm^-1^	θ_max_ = 25.2°, θ_min_ = 2.8°
ω scans	*h* = −5→6
Absorption correction: multi-scan (*ABSCOR*; Higashi, 1995)	*k* = −14→11
*T*_min_ = 0.73, *T*_max_ = 0.82	*l* = −14→14
5185 measured reflections	

### Refinement

**Table:**

Refinement on *F*^2^	Primary atom site location: structure-invariant direct methods
Least-squares matrix: full	Secondary atom site location: difference Fourier map
*R*[*F*^2^ > 2σ(*F*^2^)] = 0.035	Hydrogen site location: inferred from neighbouring sites
*wR*(*F*^2^) = 0.079	H-atom parameters constrained
*S* = 1.02	*w* = 1/[σ^2^(*F*_o_^2^) + (0.037*P*)^2^] where *P* = (*F*_o_^2^ + 2*F*_c_^2^)/3
2553 reflections	(Δ/σ)_max_ = 0.001
166 parameters	Δρ_max_ = 0.32 e Å^−^^3^
0 restraints	Δρ_min_ = −0.35 e Å^−^^3^

### Special details

**Table:**

Geometry. All e.s.d.'s (except the e.s.d. in the dihedral angle between two l.s. planes) are estimated using the full covariance matrix. The cell e.s.d.'s are taken into account individually in the estimation of e.s.d.'s in distances, angles and torsion angles; correlations between e.s.d.'s in cell parameters are only used when they are defined by crystal symmetry. An approximate (isotropic) treatment of cell e.s.d.'s is used for estimating e.s.d.'s involving l.s. planes.
Refinement. Refinement of *F*^2^ against ALL reflections. The weighted *R*-factor *wR* and goodness of fit *S* are based on *F*^2^, conventional *R*-factors *R* are based on *F*, with *F* set to zero for negative *F*^2^. The threshold expression of *F*^2^ > σ(*F*^2^) is used only for calculating *R*-factors(gt) *etc*. and is not relevant to the choice of reflections for refinement. *R*-factors based on *F*^2^ are statistically about twice as large as those based on *F*, and *R*- factors based on ALL data will be even larger.

### Fractional atomic coordinates and isotropic or equivalent isotropic displacement parameters (Å^2^)

**Table:**

	*x*	*y*	*z*	*U*_iso_*/*U*_eq_	
Br	0.29930 (7)	0.52287 (3)	0.29304 (3)	0.06212 (15)	
S1	1.14891 (18)	0.35920 (7)	−0.09979 (7)	0.0586 (2)	
S2	0.95417 (16)	0.13601 (7)	−0.02534 (7)	0.0499 (2)	
N1	0.7198 (5)	0.2821 (2)	0.11142 (18)	0.0411 (5)	
N2	0.8444 (5)	0.3398 (2)	0.04224 (18)	0.0438 (6)	
H2N	0.8360	0.4121	0.0428	0.053*	
O1	0.3795 (4)	0.0498 (2)	0.38554 (17)	0.0620 (6)	
O2	0.1403 (4)	0.1803 (2)	0.49916 (17)	0.0676 (6)	
C1	0.3386 (5)	0.3734 (3)	0.3172 (2)	0.0438 (7)	
C2	0.2229 (5)	0.3338 (3)	0.3994 (2)	0.0487 (7)	
H2	0.1332	0.3810	0.4402	0.058*	
C3	0.2405 (6)	0.2269 (3)	0.4200 (2)	0.0480 (7)	
C4	0.3727 (5)	0.1538 (3)	0.3573 (2)	0.0459 (7)	
C5	0.4848 (5)	0.1938 (3)	0.2762 (2)	0.0424 (7)	
H5	0.5721	0.1457	0.2348	0.051*	
C6	0.4722 (5)	0.3035 (2)	0.2539 (2)	0.0399 (6)	
C7	0.6023 (5)	0.3466 (3)	0.1714 (2)	0.0418 (6)	
H7	0.6001	0.4227	0.1621	0.050*	
C8	0.9769 (5)	0.2856 (2)	−0.0251 (2)	0.0392 (6)	
C9	1.1501 (7)	0.0933 (3)	−0.1230 (3)	0.0603 (9)	
H9A	1.0899	0.1146	−0.1923	0.090*	
H9B	1.1296	0.0081	−0.1393	0.090*	
H9C	1.3358	0.1345	−0.0899	0.090*	
C10	0.5363 (7)	−0.0214 (3)	0.3356 (3)	0.0635 (9)	
H10A	0.4618	−0.0497	0.2548	0.095*	
H10B	0.5356	−0.0888	0.3675	0.095*	
H10C	0.7174	0.0263	0.3506	0.095*	
C11	0.0060 (7)	0.2495 (4)	0.5675 (3)	0.0780 (12)	
H11A	0.1296	0.3245	0.6114	0.117*	
H11B	−0.0597	0.2057	0.6179	0.117*	
H11C	−0.1419	0.2648	0.5192	0.117*	

### Atomic displacement parameters (Å^2^)

**Table:**

	*U*^11^	*U*^22^	*U*^33^	*U*^12^	*U*^13^	*U*^23^
Br	0.0681 (3)	0.0539 (2)	0.0700 (2)	0.02466 (17)	0.02423 (18)	0.01301 (17)
S1	0.0871 (6)	0.0518 (5)	0.0646 (5)	0.0304 (4)	0.0508 (5)	0.0325 (4)
S2	0.0635 (5)	0.0409 (4)	0.0588 (5)	0.0197 (4)	0.0304 (4)	0.0217 (4)
N1	0.0497 (14)	0.0445 (14)	0.0364 (12)	0.0116 (11)	0.0202 (11)	0.0177 (11)
N2	0.0615 (16)	0.0401 (13)	0.0443 (13)	0.0190 (12)	0.0286 (12)	0.0215 (11)
O1	0.0794 (16)	0.0694 (15)	0.0599 (13)	0.0274 (12)	0.0413 (12)	0.0352 (12)
O2	0.0721 (15)	0.0933 (17)	0.0550 (13)	0.0250 (13)	0.0420 (12)	0.0298 (12)
C1	0.0391 (16)	0.0493 (17)	0.0406 (15)	0.0114 (13)	0.0083 (13)	0.0083 (13)
C2	0.0431 (17)	0.070 (2)	0.0359 (15)	0.0200 (15)	0.0177 (13)	0.0067 (15)
C3	0.0424 (17)	0.070 (2)	0.0361 (15)	0.0117 (15)	0.0180 (13)	0.0177 (15)
C4	0.0435 (17)	0.0566 (19)	0.0369 (15)	0.0064 (14)	0.0137 (13)	0.0139 (14)
C5	0.0459 (17)	0.0515 (18)	0.0346 (14)	0.0148 (14)	0.0190 (13)	0.0112 (13)
C6	0.0382 (15)	0.0470 (17)	0.0354 (14)	0.0094 (13)	0.0133 (12)	0.0106 (13)
C7	0.0481 (17)	0.0421 (16)	0.0391 (15)	0.0117 (13)	0.0159 (13)	0.0142 (13)
C8	0.0474 (16)	0.0425 (16)	0.0346 (14)	0.0158 (13)	0.0144 (13)	0.0166 (12)
C9	0.073 (2)	0.0517 (19)	0.073 (2)	0.0287 (17)	0.0390 (18)	0.0213 (17)
C10	0.083 (2)	0.062 (2)	0.064 (2)	0.0289 (19)	0.0369 (19)	0.0264 (17)
C11	0.067 (2)	0.124 (3)	0.0492 (19)	0.024 (2)	0.0377 (18)	0.016 (2)

### Geometric parameters (Å, °)

**Table:**

Br—C1	1.893 (3)	C3—C4	1.411 (4)
S1—C8	1.662 (3)	C4—C5	1.376 (4)
S2—C8	1.741 (3)	C5—C6	1.390 (4)
S2—C9	1.789 (3)	C5—H5	0.9300
N1—C7	1.280 (3)	C6—C7	1.454 (4)
N1—N2	1.381 (3)	C7—H7	0.9300
N2—C8	1.328 (3)	C9—H9A	0.9600
N2—H2N	0.8600	C9—H9B	0.9600
O1—C4	1.349 (3)	C9—H9C	0.9600
O1—C10	1.421 (4)	C10—H10A	0.9600
O2—C3	1.360 (3)	C10—H10B	0.9600
O2—C11	1.431 (4)	C10—H10C	0.9600
C1—C2	1.395 (4)	C11—H11A	0.9600
C1—C6	1.398 (4)	C11—H11B	0.9600
C2—C3	1.355 (4)	C11—H11C	0.9600
C2—H2	0.9300		
			
C8—S2—C9	101.93 (13)	N1—C7—C6	121.3 (3)
C7—N1—N2	113.4 (2)	N1—C7—H7	119.3
C8—N2—N1	121.0 (2)	C6—C7—H7	119.3
C8—N2—H2N	119.5	N2—C8—S1	120.9 (2)
N1—N2—H2N	119.5	N2—C8—S2	114.23 (19)
C4—O1—C10	118.1 (2)	S1—C8—S2	124.83 (16)
C3—O2—C11	117.8 (3)	S2—C9—H9A	109.5
C2—C1—C6	120.9 (3)	S2—C9—H9B	109.5
C2—C1—Br	117.3 (2)	H9A—C9—H9B	109.5
C6—C1—Br	121.8 (2)	S2—C9—H9C	109.5
C3—C2—C1	120.3 (3)	H9A—C9—H9C	109.5
C3—C2—H2	119.8	H9B—C9—H9C	109.5
C1—C2—H2	119.8	O1—C10—H10A	109.5
C2—C3—O2	125.5 (3)	O1—C10—H10B	109.5
C2—C3—C4	120.3 (2)	H10A—C10—H10B	109.5
O2—C3—C4	114.2 (3)	O1—C10—H10C	109.5
O1—C4—C5	126.1 (3)	H10A—C10—H10C	109.5
O1—C4—C3	115.1 (2)	H10B—C10—H10C	109.5
C5—C4—C3	118.7 (3)	O2—C11—H11A	109.5
C4—C5—C6	122.3 (3)	O2—C11—H11B	109.5
C4—C5—H5	118.9	H11A—C11—H11B	109.5
C6—C5—H5	118.9	O2—C11—H11C	109.5
C5—C6—C1	117.5 (2)	H11A—C11—H11C	109.5
C5—C6—C7	121.5 (2)	H11B—C11—H11C	109.5
C1—C6—C7	121.0 (3)		

### Hydrogen-bond geometry (Å, °)

**Table:**

*D*—H···*A*	*D*—H	H···*A*	*D*···*A*	*D*—H···*A*
N2—H2N···S1^i^	0.86	2.62	3.456 (4)	166

Symmetry codes: (i) −*x*+2, −*y*+1, −*z*.
